# Preparing for future pandemics: A qualitative exploration of social media in light of the COVID-19 pandemic and vaccine hesitancy

**DOI:** 10.1371/journal.pgph.0004317

**Published:** 2025-07-07

**Authors:** Emmanuel A. Odame, Oluwabunmi Dada, Jordan Nelson, Ayorinde Ogunyiola, Jessica Haley

**Affiliations:** 1 Department of Environmental Health Sciences, School of Public Health, University of Alabama at Birmingham, Birmingham, Alabama, United States of America; 2 Department of Occupational Safety and Health, Murray State University, Murray, Kentucky, United States of America; 3 Independent Researcher, Birmingham, Alabama, United States of America; 4 Department of Political Science & Sociology, Murray State University, Murray, Kentucky, United States of America.; New York University Grossman School of Medicine, UNITED STATES OF AMERICA

## Abstract

Vaccine hesitancy remains a significant barrier to the success of global vaccination campaigns and vaccine programs. Understanding people’s perceptions of vaccines on social media during disease outbreaks can aid in reducing socially induced vaccine hesitancy and improve program implementation. Social media is an increasingly valuable tool for assessing public perceptions on critical issues, including vaccine adoption. This study examines perceptions surrounding the COVID-19 vaccine among Twitter users in the United States, Brazil, and India within a few weeks post-vaccine release. These countries are associated with anti-vaccine movements and outbreaks of vaccine-preventable diseases. We identified and analyzed key themes related to vaccine perception in 2,858 Twitter posts. Using a qualitative interpretive approach to analyze tweets, we found that mistrust in vaccine science, politics of vaccination, and religious pushbacks were the main themes that emerged from the analysis. Perceptions harbored by individuals and communicated frequently via mass communication platforms may erode public trust and disarticulate avenues of communication between public health officials and communities. Thus, we suggest that harnessing vaccine hesitancy-related information on social media can enhance understanding of public perceptions about vaccines while providing opportunities for interventional communications to educate the public.

## Introduction

There is no doubt that vaccination is one of the most outstanding achievements of Public Health [1]. It is one of the most cost-effective public health measures, saving millions of lives from infectious diseases, including COVID-19 [2,3]. Nonetheless, vaccine hesitancy remains a top threat to global health [3], becoming more evident during the COVID-19 pandemic [4]. Reasons for vaccine hesitancy may be complex, involving political, social, and behavioral factors [3–9]. However, there is evidence that complacency, inconvenience in accessing vaccines, lack of confidence in vaccines, and mistrust fueled by misinformation are key contributors [3,6].

The internet, including social media, is a well-established communication tool currently recognized as a social determinant of health [10,11]. Social media can be a risk communication tool for disaster planning, response, and research [12,13]. Its versatility in real-time communication and active engagement makes it more attractive to a larger audience than traditional platforms such as radio and television [14,15].

Additionally, it is utilized for health promotion initiatives such as addressing vaccine concerns and increasing immunization rates [16–19]. However, it can also serve as a source of vaccine misinformation, particularly where intensifying perceptions of vaccination mistrust can spread [20]. Evidence indicates that anti-vaccination movements have become particularly influential on this platform due to their potential to reach millions of people instantaneously [7,8]. The use of social media is also known to contribute significantly to low adherence to vaccination due to misinformation, leading to poor health literacy [21].

The COVID-19 pandemic presented devastating impacts on human lives and livelihoods, with ripple effects on healthcare, food, and other systems. In fact, the pandemic has caused at least 18.2 million deaths, plunged millions into poverty, and further exacerbated existing disparities in health outcomes [22,23]. Although COVID-19 had a global impact, the United States (U.S.), Brazil, and India reported the highest numbers of cases and deaths related to the virus [24,25]. Despite efforts by governments and experts to embark on mass vaccination rollouts, vaccination coverages in these countries were below the herd immunity threshold [26]. Thus, an insight into ongoing discussions or themes on social media can inform strategies to improve vaccine uptake. In general, a high acceptance rate of the vaccine is crucial for achieving sufficient immunization coverage and protecting the public’s health [27]. Examining perceptions on vaccinations and reasons why people may or may not want to vaccinate can be helpful for health experts and policymakers to respond more rapidly based on empirical evidence [28].

Against this background, our study investigated the perceptions surrounding the COVID-19 vaccine among Twitter users in the countries most affected by the COVID-19 pandemic, the U.S., Brazil, and India, during the week immediately after the vaccine release. The question motivating this study is: *what are the public perceptions of Twitter users on COVID-19 vaccination in the U.S., Brazil, and India between December 2020 and February 2021?* We analyzed emerging social media themes on vaccination decisions in these countries, which can inform preparations for future pandemics and other public health crises.

## Materials and methods

### 2.1. Data collection

We collected data from Twitter (currently rebranded as X) through Meltwater (www.Meltwater.com) software, a dedicated online social media analysis platform that extracts tweets in a Boolean search style [29]. The study utilized Twitter because of its standard word limit of 280 characters, which concisely captures users’ expressions compared to other social media platforms [30]. Twitter provides real-time insights into public discourse, behaviors, and trends across various topics and demographics. The platform’s accessible API allows for the collection of user-generated content, such as text, hashtags, and metadata, which are invaluable for analyzing social dynamics, public sentiment, and emerging issues important in public health research [31]. Twitter’s global reach and immediacy makes it an ideal platform for studying phenomena that require current and geographically diverse insights. To begin retrieving data from Meltwater, we developed keywords that reflected the goals of this study. A combination of keywords included are coronavirus, vaccines, Pfizer-BioTech, Moderna, Johnson & Johnson/Jassen, AstraZeneca, Novavax, Sinovac-Biotech, Covaxin, Covishield, Sputnik, United States, Brazil, and India in our search query. We combined keywords in a Boolean search style (i.e., COVID-19 and Pfizer) to retrieve relevant social media posts on COVID-19 vaccination and vaccines. Combining these keywords provides a vast expanse of perspectives from social media users on COVID-19 vaccination. We provide a brief description of these keywords (Table 1).

**Table 1:** Keywords and their explanations.

Keywords	Description
“Coronavirus” OR “COVID*” OR “Corona*” AND “Vaccin*”	Provide information generally about social media posts on coronavirus and vaccination
“Pfizer-BioTech” OR “Moderna” OR “Johnson & Johnson” OR “AstraZeneca” OR “Novavax” OR “Sinovac-BioTech” OR “Covaxin OR “Covishield OR “Sputnik”	Represent the vaccines administered in different countries (United States, Brazil, and India)
“United States” “U.S.*” OR “Brazil” OR “India”	Captures countries included in the study (United States, Brazil, and India)

*represents Boolean search style for individual words (e.g., Vaccin* = vaccines, vaccine, vaccination)

### 2.2. Inclusion and exclusion criteria

The inclusion criteria focused on tweets written in English between December 2020 and February 2021, discussing COVID-19 vaccination specifically within the United States, Brazil, or India. Tweets falling outside this time frame, not in English, or unrelated to COVID-19 vaccination in the specified countries were excluded to ensure data relevance and consistency. Table 2 provides the criteria for selecting tweets included and excluded in the analysis.

**Table 2:** Study inclusion and exclusion criteria.

S/N	Inclusion criteria	Exclusion criteria
1	Tweets written within the period December 2020-February 2021	Exclude tweets not written within the time frame December 2020-February 2021
2	Include tweets that discuss COVID-19 vaccination	Exclude tweets not discussing COVID-19 Vaccination
3	Include tweets only focusing on discussing COVID-19 vaccination in the United States, Brazil, or India	Exclude tweets not focusing on discussing COVID-19 vaccination in the United States, Brazil, or India
4	Include only English language tweets during the study period	Exclude tweets not written in English language

### 2.3. Analytical approach and data analysis

Before we analyzed Twitter data, search query results from Meltwater were exported into a repository and rearranged into the following categories: date, country, Twitter usernames, and tweets. R software was utilized to remove usernames, web links, and emojis. Once data was organized and stripped of personal information, Nvivo 12 statistical software was employed to interpret tweets and draw meanings through qualitative interpretative analysis. This interpretative approach has been successfully used to explore views about social reality [32,33]. Themes were not determined a priori; instead, we allowed themes to emerge from our data. This interpretive approach ensures that the meaning associated with social phenomena is constructed [32,33], and builds on the fact that the social world has diverse interpretations [33]. The interpretive approach contrasts statistical techniques employed in positivist research. Analytical rigor from interpretive methods emerges from the systematic and transparent data collection and analysis method rather than statistical inferences for constructing validity [34].

In analyzing Twitter data through a qualitative interpretative approach, we manually coded 10% of the tweets from each country (United States n = 238; Brazil n = 84; India n = 40) in NVivo, organizing them into emerging themes and subthemes. This manual coding process was essential to ensure the quality and validity of the analysis. It allowed us to familiarize ourselves with the data, identify key themes, and develop a consistent coding framework aligned with the research objectives. The manual coding process also helped calibrate the auto-coding algorithm to meet the specific needs of the study, minimizing potential biases or inaccuracies in automated classifications [35]. During the manual coding in Nvivo, we marked portions of tweets that referred to user perceptions of the COVID-19 vaccine and interpreted the excerpts using a structured process. First, we classified each tweet as relevant to specific codes, and next, we deduced the underlying meaning or reference of each tweet. While analyzing the data, we considered the final themes that emerged from our interpretation: politics, trust in vaccine science, religious pushbacks, misinformation about COVID-19 vaccination, and other related subthemes.

We developed a codebook (see S1 Table) to guide our coding process. After coding 10 percent of the data for each country, we used the auto code function in Nvivo 12 to code the rest of the data automatically, identifying statements and sentences that answered the question we asked. This resulted in a total of 2,858 Tweets included in the analysis (United States n = 2,380; Brazil n = 83; India n = 395). To finalize our coding process, we arranged codes into themes and sub-themes based on interpretations and meanings that emerged from the data, categorizing them by themes that reveal the perception of Twitter users in the U.S., Brazil, and India.

### 2.4. Ethical consideration

The data used in this study were collected and analyzed in accordance with the terms and conditions set by Twitter ensuring compliance with all relevant ethical requirements [36]. The study adhered to ethical principles and did not require an Institutional Review Board (IRB) or ethics committee approval, as we used publicly available data (tweets) without involving human participants. Similarly, we followed previous studies such as Saleh et al., 2023, Chen et al., 2020, and Sousa‐Pinto et al., 2024, which analyzed tweets by stripping personal information while presenting only relevant portions of the tweets, ensuring that individual accounts could not be identified [37–39]. Informed consent was not applicable since no direct interaction with participants occurred [37–40].

By focusing on tweets, we ensured sufficient rigor in evaluating perceptions of COVID-19 within this study. However, our approach may have excluded relevant tweets and perspectives from the countries analyzed. The final dataset consists solely of tweets written and published in English. While these decisions align with commonly accepted scholarly standards and practical considerations, we recognize that they may have constrained the scope of our study.

## Results

### 3.1. Metadata for themes discussed by Twitter users in the U.S., Brazil, and India

**Fig 1:**
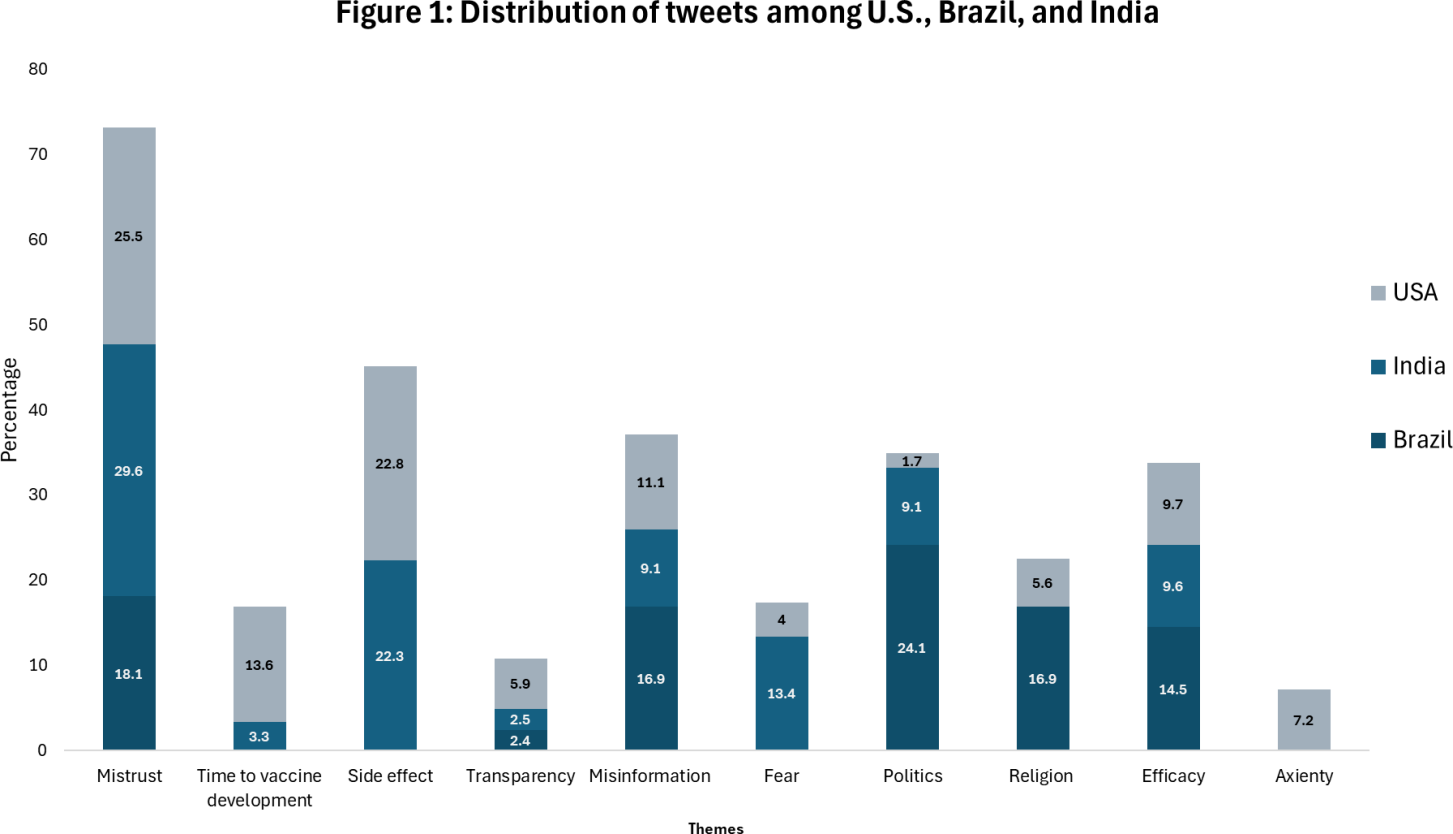
Distribution of tweets among U.S., Brazil, and India.

In this section, we describe themes that emerged from analyzing Twitter data to answer the question: *what are the public perceptions of Twitter users on COVID-19 vaccination in the U.S., Brazil, and India between December 2020 and February 2021?* Using an interpretative approach detailed above, we identify three key themes that emerged from our analysis of Twitter data: (1) Mistrust in vaccine science, (2) politics of vaccination, and (3) religious pushbacks. Table 3 presents the themes and subthemes that emerged. Fig 1 presents metadata for unique tweets in each theme that merged through the coding process.

**Table 3:** An overview of themes and subthemes from twitter data in the U.S., Brazil, and India.

	United States *Frequency of Count (%)	Brazil *Frequency of Count (%)	India *Frequency of Count (%)	Total *Frequency of Count (%)
**Mistrust in vaccine science**	**2,207 (92.7)**	**49 (59.0)**	**359 (90.9)**	**2,615 (91.5)**
General lack of trust in vaccines	608 (25.55)	15 (18.07)	117 (29.62)	740 (25.89)
Time to vaccine development	324 (13.61)	---	13 (3.29)	337 (11.79)
Unknown side effects and potentially creating new disease	542 (22.77)	---	92 (23.29)	634 (22.18)
Transparency in vaccine development	140 (5.88)	2 (2.41)	10 (2.53)	152 (5.32)
Misinformation	265 (11.13)	14 (16.86)	36 (9.11)	315 (11.02)
Fear	96 (4.03)	---	53 (13.42)	149 (5.21)
Efficacy	232 (9.75)	12 (14.45)	38 (9.62)	282 (9.87)
Anxiety	---	6 (7.23)	---	6 (0.21)
**Politics of vaccination**	**40 (1.7)**	**20 (24.1)**	**36 (9.1)**	**96 (3.4)**
**Religious Push backs**	**133 (5.6)**	**14 (16.9)**	**---**	**145 (5.1)**
**Total Tweets included**	**2,380**	**83**	**395**	**2,858**

We found similarities and differences in themes on the perception of COVID-19 vaccines in the U.S., Brazil, and India. Mistrust in vaccine science was the most discussed theme by Twitter users in the U.S., Brazil, and India between December 2020 and February 2021. A second theme commonly discussed was religious pushback on vaccination. The differences in themes that emerged were regarding vaccine development in the U.S. and the distribution of vaccines in India and Brazil. We grouped sub-themes that emerged into large themes, as explained in Table 3. The following subsections expand on each perception held by Twitter users.

### 3.2. Emerging perception of COVID-19 vaccination

#### 3.2.1. Mistrust in vaccine science.

A common perception expressed by Twitter users on COVID-19 that emerged from tweets in the U.S., Brazil, and India was a lack of trust (mistrust) in vaccine science. This perception accounted for the vast majority of Twitter posts in the U.S. (92.73%), Brazil (59.03%), and India (90.9%). Of the subthemes identified, concerns of general mistrust in the vaccine (25.89%) and the potential for unknown side effects or the creation of a new disease (22.18%) were the most prevalent. In contrast, posts regarding the transparency of vaccine development (5.32%), fear (5.21%), and anxiety (0.21%) accounted for the smallest number of posts.

Twitter users expressed skepticism regarding the development of COVID-19 vaccines and its potential for unknown side effects on their health. Many Twitter users expressed a lack of trust in the vaccine due to the vaccine’s rapid development and distribution to the public. Twitter users posted statements on the unknown effectiveness of these vaccines since COVID-19 vaccines were developed in less than a year. For instance, a user opined in Brazil that, “There is not enough data about the effectiveness of the AstraZeneca vaccine for patients over 65 years of age.” The lack of data to test for efficacy over a long period and significant mistrust of vaccines appeared to create skepticism among Twitter users. Users were also concerned that taking the vaccine could lead to adverse health outcomes such as allergic response, infertility, comorbid diseases, and transgenerational disease development.

Concerns regarding the lack of transparency, fear, and anxiety emerged as additional key sub-themes. In the U.S., approximately 140 unique tweets suggest that tweet users believe there is a lack of transparency on how the vaccines were developed. Brazil and India had significantly fewer tweets regarding concerns about vaccine transparency (n = 12). Tweets categorized in the sub-themes of fear and anxiety had the lowest frequency of 5.42% combined. For instance, a Twitter user from the U.S. asserts, “What if this COVID vaccine is what starts off a zombie apocalypse? The world we live in: A year and a half from now, when the vaccine has kicked in, when COVID cases become rare, the pandemic deniers will say... see, we knew it was a hoax.”

Tweets regarding vaccine misinformation were further evident among Twitter users. For instance, a Twitter user in the U.S. asserts, “Actually, a double dose of [Bacillus Calmette-Guerin] BCG vaccine clubbed in one is the best medicine to fight COVID- 19...see we have to understand this COVID is virus and BCG has the power to fight all viruses if made more powerful without hurting the RNA which this Pfizer vaccine will do.”

#### 3.2.2. Politics of vaccination.

The subject matter of the politics of vaccines was apparent, with varying opinions among the countries we studied. There is a large consensus that the development and administration of vaccines are politically motivated, according to Twitter posts. For instance, a Twitter user from the U.S. posted, “The vaccine carries more risk than benefit, to date, politics has played as big a role in as many aspects involving the infection, as ever before.” Other Twitter users further express the political influence of vaccine acceptance or distrust based on differences in the political party stance. For instance, a Twitter user expressed: “If COVID-19 is a hoax made up by Democrats to make Trump look bad (as if...), why are the republicans getting vaccinated against it?” Twitter users in Brazil shared similar perceptions. In Brazil, Twitter users express displeasure on how governments and leaders have managed COVID-19 and vaccine distribution. For instance, a Twitter user opined: “Governments the world over appears to have underestimated the complexity of the logistics around COVID-19.” There were also concerns that politicians do not fully communicate the facts about COVID-19, as a Twitter user expressed that authorities are “hiding the real data of COVID vaccine” in Brazil.

#### 3.2.3. Religious based perception.

Religious perceptions were only reported in the U.S. and Brazil, accounting for 5.1% of Twitter posts. Noteworthy is the influence of religious leaders on their followers, which can either promote distrust or discouragement of the use of COVID-19 vaccines. For instance, “In the Brazilian state of Amazonas, leaders of the Kokama indigenous tribe are complaining that evangelical pastors are trying to talk them out of taking the COVID-19 vaccine.” Opinions formed by religious leaders influence the congregation, which ultimately creates a perception that religious followers have concerning vaccine acceptance. With this opinion formed, Twitter users now believe that “the coronavirus is widespread in the world, even with the best vaccines, it will take us years to eradicate the virus if there are no other variants; there are more than seven billion people in the world, and each nation with its methods of fighting the virus; it is better to trust God.” There is a possibility that public perceptions, including a lack of trust in vaccines and politically formed beliefs, can further exacerbate the goal of eradicating COVID-19 globally.

## Discussion

This study describes social media themes from the U.S., Brazil, and India during the timeframe immediately post-vaccine release for each country. These countries have contributed significantly to the global COVID-19 burden due to high numbers of cumulative excessive deaths. First and foremost, our findings indicate that the perception held by Twitter users is mainly associated with mistrust in vaccine science due to (1) a general lack of trust in vaccines and (2) unknown possible side effects and potentially creating new diseases. Concerns about vaccine safety are as old as vaccines and pre-existed COVID-19 [41]. Thus, trust or lack of confidence in vaccines remains the most crucial underlying reason in vaccination decisions, the most common theme in all three countries. In assessing trust in the COVID-19 vaccine, Latkin et al. reported vaccine fast-tracking and no knowledge of long-term side effects were the two major concerns among the public [42]. Evidence suggests strong confidence in COVID-19 vaccines leads to higher vaccination coverage [43]. Hence, the proportion of the vaccinated population increases when there is a decrease in those who doubt or have safety concerns and would not want to vaccinate [44]. A study by Daly et al. observed an increase in public trust in vaccination from October 2020 to March 2021; however, a substantial proportion of the population remained skeptical about the vaccine [45]. Data from the Centers for Disease Control and Prevention (CDC) similarly indicates that a substantial proportion of U.S. adults will probably not get vaccinated, including those who have not yet decided [43]. Thus, rebuilding and sustaining public trust is necessary to increase vaccination coverage. An excellent approach to rebuilding trust is by conducting a complete assessment of the community’s history, socioeconomic background, previous experience, views of those they trust and distrust, and how informed they are about the benefits and risks of vaccines [41]. Public concerns about vaccines are vital to successful disease mitigation and must not be dismissed. Health officials should make conscientious efforts to understand the public’s perceptions and, as much as possible, incorporate the perspectives of the community in planning vaccine policies and programs.

Our study also highlights that religious pushback was more commonly discussed in the U.S. and Brazil than in India. Religion is known to influence vaccination decisions [46], where refusal of vaccines has also been linked with strong religious convictions. Religious objections to vaccines are broadly based on two tenets: the ethical dilemmas associated with using cells to create vaccines and beliefs that the body is sacred, should not receive certain chemicals or blood or tissues from animals, and should be healed by God or natural means [47]. For instance, certain religious communities are well-known for rejecting vaccination for religious reasons [6,46,48,49]. A study reported that parents with frequent attendance at religious services were more likely to refuse vaccination than those who did not regularly attend service [50]. Public health experts have to recognize that religious and traditional leaders are critical in vaccine promotion [51]. This is because they are highly esteemed, considered trustworthy, and greatly influence their members’ decisions [48,52]. A qualitative study by Ruijs et al. examined the role of religious leaders in promoting the acceptance of vaccines and reported that most leaders support voluntary vaccination programs and religious exemptions from these programs [48]. Additionally, these leaders emphasized their willingness to have a dialogue with authorities even though they are not likely to promote vaccines on behalf of authorities. The inclusion of traditional and religious leaders in vaccine conversations has the potential to impact perceptions among their followers significantly.

The role of politics in vaccination is known among public health officials to contribute significantly to public perception of vaccines. The extent of political influence surrounding vaccination differs from region to region [53,54]. For instance, political polarization has been reported to influence the vaccination rate in the U.S. [55]. At the same time, Brazil faces a lack of coordination between the federal and state governments [55,56], and India has distribution and administration challenges due to the country’s large population [57]. Despite these various assertions, our study found similar perceptions across these countries. Political influence on vaccination predates now [54]; yellow fever vaccination in Brazil was not easy, as this was a significant public health challenge for almost a century [54]. Similarly, in India, Mahatma Gandhi, who was then the leading representative of the Indian nation, articulated his reservations against vaccination due to his religious and cultural (personal) beliefs, which led to some Indians rejecting vaccination at the time [54]. Vaccination is an essential tool that public health uses to curb the spread of diseases and thus should be strongly encouraged, irrespective of political views and affiliations.

Perceptions centering on political ambitions can be catastrophic to ensuring the population’s safety [58]. It is evident that political polarization in the U.S. hampers vaccination against COVID-19 [55], as vaccine administration data revealed a disparity in counties that favor one political party over another [59]. Moreover, a longitudinal study of U.S. residents conducted before vaccines were approved reported a decline in attitude towards getting a vaccine when one becomes available [4]. Furthermore, another study examined the impact of vaccine approval relative to the U.S. election and government officials’ vaccine endorsement on vaccine hesitancy among Americans. Their results showed that announcing the vaccine one week before the election significantly reduced confidence in the vaccine, and the endorsement of one of the government leaders, who is one of the leading experts in the U.S. on infectious diseases, increased confidence in the vaccine and the willingness to get vaccinated [60]. Before vaccines were approved in the U.S., Americans had concerns about whether vaccine approval would be based on safety and effectiveness rather than political ambitions [55,60,61]. A continuous political divide can impede public health’s concept of vaccination, which may be critical in preventing other outbreaks in the future [55]. Carefully communicating the science behind the vaccination process may help policymakers see an improvement in public support towards vaccine uptake [62].

At the start of COVID-19, Brazil lacked agreement between the federal and state governments on guidelines to curb the spread; the state took quick actions and measures, while the federal government was more concerned about the economy and discredited vaccination programs [63,64]. This dispute led to government officials being dismissed due to diverse opinions from the federal government, which might have affected vaccine acceptance despite the country’s excellent immunization campaign record prior [64]. A study examined how Brazil’s political climate affected vaccine acceptance based on the country of vaccine production and found that vaccines from China and Russia were mostly rejected by Brazilians affiliated with the incumbent political party [65]. Public health should not be based on political opinions and affiliations, which may significantly influence people’s perceptions and exacerbate the impact of the pandemic [65,66].

To a large extent, India had a robust plan for COVID-19 vaccine production, not only for domestic use but also for distribution across nations that can’t afford expensive vaccines produced in developed countries [57,67]. India is considered one of the world’s largest vaccines manufacturers including the production of COVID-19 vaccines [67–69]. Challenges, however, such as stocking and distribution coupled with restrictions on access to raw materials for vaccine production resulted in an unsuccessful domestic vaccination rollout [68]. Of note, despite these domestic challenges, vaccines were still exported until later when the Government of India temporarily halted all exports of the Covishield vaccine to address local needs [68]. Moreover, the emergency approval of the first two vaccines, Covishield and Covaxin, has been criticized and said to be due to nationalistic political forces [67]. This underscores the need for sufficient time for clinical trials and ascertaining each vaccine’s effectiveness and accompanying side effects. All these raised questions on India’s regulatory dependability, which may, in turn, affect public perception of vaccination.

## Conclusion

Understanding individual perceptions regarding vaccine acceptance is critical for successful vaccine campaigns. Notably, rapid identification of themes in public perception may be advantageous in informing health officials and developing effective communications targeted at the public’s specific concerns. In this study, we identified the common themes in Twitter posts at the early stages of vaccine public availability in the U.S., Brazil, and India. The common perceptions identified, such as a lack of scientific or political transparency, can damage trust and disarticulate avenues of communication between public health officials and communities. Social media platforms provide a real-time understanding of public perceptions about vaccines, enhancing opportunities for interventional communications to educate the public. Future work should incorporate how media-based public perceptions change over time and how communication intervention campaigns influence these perceptions.

## Supporting information

S1 Table Codebook for Preparing for Future Pandemics: A qualitative exploration of social media in light of the COVID-19 pandemic and vaccine hesitancy. (DOCX)

S2 Table Study inclusion and exclusion criteria. (DOCX)

## References

[1] Centers for Disease Control and Prevention (CDC). Ten great public health achievements--worldwide, 2001-2010. MMWR Morb Mortal Wkly Rep. 2011;60(24):814–8. 21697806

[2] Andre FE, Booy R, Bock HL, Clemens J, Datta SK, John TJ, et al. Vaccination greatly reduces disease, disability, death and inequity worldwide. Bull World Health Organ. 2008;86(2):140–6. doi: 10.2471/blt.07.040089 18297169 PMC2647387

[3] WHO. Ten threats to global health in 2019. World Health Organization. 2019. https://www.who.int/news-room/spotlight/ten-threats-to-global-health-in-2019

[4] Fridman A, Gershon R, Gneezy A. COVID-19 and vaccine hesitancy: A longitudinal study. PLoS One. 2021;16(4):e0250123. doi: 10.1371/journal.pone.0250123 33861765 PMC8051771

[5] Dubé È, Farrands A, Lemaitre T, Boulianne N, Sauvageau C, Boucher FD, et al. Overview of knowledge, attitudes, beliefs, vaccine hesitancy and vaccine acceptance among mothers of infants in Quebec, Canada. Hum Vaccin Immunother. 2019;15(1):113–20. doi: 10.1080/21645515.2018.1509647 30095325 PMC6363056

[6] Dubé E, Laberge C, Guay M, Bramadat P, Roy R, Bettinger J. Vaccine hesitancy: an overview. Hum Vaccin Immunother. 2013;9(8):1763–73. doi: 10.4161/hv.24657 23584253 PMC3906279

[7] Dubé E, Vivion M, MacDonald NE. Vaccine hesitancy, vaccine refusal and the anti-vaccine movement: influence, impact and implications. Expert Rev Vaccines. 2015;14(1):99–117. doi: 10.1586/14760584.2015.964212 25373435

[8] Dubé È, Ward JK, Verger P, MacDonald NE. Vaccine Hesitancy, Acceptance, and Anti-Vaccination: Trends and Future Prospects for Public Health. Annu Rev Public Health. 2021;42:175–91. doi: 10.1146/annurev-publhealth-090419-102240 33798403

[9] Hall R. Anti-vaccination movement could derail fight against coronavirus, experts warn. Independent. https://www.the-independent.com/news/world/americas/coronavirus-vaccine-anti-vaxxer-donald-trump-a9426151.html 2020.

[10] Morley J, Cowls J, Taddeo M, Floridi L. Public Health in the Information Age: Recognizing the Infosphere as a Social Determinant of Health. J Med Internet Res. 2020;22(8):e19311. doi: 10.2196/19311 32648850 PMC7402642

[11] Benis A, Khodos A, Ran S, Levner E, Ashkenazi S. Social Media Engagement and Influenza Vaccination During the COVID-19 Pandemic: Cross-sectional Survey Study. J Med Internet Res. 2021;23(3):e25977. doi: 10.2196/25977 33651709 PMC7968480

[12] Chan JL, Purohit H. Challenges to Transforming Unconventional Social Media Data into Actionable Knowledge for Public Health Systems During Disasters. Disaster Med Public Health Prep. 2020;14(3):352–9. doi: 10.1017/dmp.2019.92 31610817

[13] Houston JB, Hawthorne J, Perreault MF, Park EH, Goldstein Hode M, Halliwell MR, et al. Social media and disasters: a functional framework for social media use in disaster planning, response, and research. Disasters. 2015;39(1):1–22. doi: 10.1111/disa.12092 25243593

[14] Betsch C, Brewer NT, Brocard P, Davies P, Gaissmaier W, Haase N, et al. Opportunities and challenges of Web 2.0 for vaccination decisions. Vaccine. 2012;30(25):3727–33. doi: 10.1016/j.vaccine.2012.02.025 22365840

[15] Buller DB, Walkosz BJ, Berteletti J, Pagoto SL, Bibeau J, Baker K, et al. Insights on HPV vaccination in the United States from mothers’ comments on Facebook posts in a randomized trial. Hum Vaccin Immunother. 2019;15(7–8):1479–87. doi: 10.1080/21645515.2019.1581555 30785361 PMC6746513

[16] Daley MF, Narwaney KJ, Shoup JA, Wagner NM, Glanz JM. Addressing Parents’ Vaccine Concerns: A Randomized Trial of a Social Media Intervention. Am J Prev Med. 2018;55(1):44–54. doi: 10.1016/j.amepre.2018.04.010 29773490 PMC8606186

[17] Glanz JM, Wagner NM, Narwaney KJ, Kraus CR, Shoup JA, Xu S, et al. Web-based Social Media Intervention to Increase Vaccine Acceptance: A Randomized Controlled Trial. Pediatrics. 2017;140(6):e20171117. doi: 10.1542/peds.2017-1117 29109107 PMC8574135

[18] Coomes EA, Haghbayan H, Finken LR, Quadros KK, Bagai A, Cheema AN. Information on Cardiovascular Disease in the Digital Era: Results From a Cross-Sectional Patient Survey. Can J Cardiol. 2019;35(6):791–4. doi: 10.1016/j.cjca.2019.03.015 31151715

[19] Gunaratne K, Haghbayan H, Coomes EA. Tweeting Authors: Impact on Research Publicity and Downstream Citations. J Gen Intern Med. 2020;35(6):1926–7. doi: 10.1007/s11606-019-05454-0 31654356 PMC7280412

[20] Faasse K, Chatman CJ, Martin LR. A comparison of language use in pro- and anti-vaccination comments in response to a high profile Facebook post. Vaccine. 2016;34(47):5808–14. doi: 10.1016/j.vaccine.2016.09.029 27707558

[21] Ashkenazi S, Livni G, Klein A, Kremer N, Havlin A, Berkowitz O. The relationship between parental source of information and knowledge about measles / measles vaccine and vaccine hesitancy. Vaccine. 2020;38(46):7292–8. doi: 10.1016/j.vaccine.2020.09.044 32981777

[22] DeSalvo K, Hughes B, Bassett M, Benjamin G, Fraser M, Galea S, et al. Public Health COVID-19 Impact Assessment: Lessons Learned and Compelling Needs. NAM Perspect. 2021;2021:10.31478/202104c. doi: 10.31478/202104c 34532688 PMC8406505

[23] The Lancet Public Health. COVID-19 pandemic: what’s next for public health?. Lancet Public Health. 2022;7(5):e391. doi: 10.1016/S2468-2667(22)00095-0 35487223 PMC9042208

[24] WHO. WHO Coronavirus (COVID-19) Dashboard. https://covid19.who.int/. 2021. 2021 June 5

[25] WHO. Coronavirus (COVID-19) vaccinations. https://ourworldindata.org/covid-vaccinations. 2021. 2021 June 18

[26] Aschwanden C. Five reasons why COVID herd immunity is probably impossible. Nature. 2021;591(7851):520–2. doi: 10.1038/d41586-021-00728-2 33737753

[27] Solís Arce JS, Warren SS, Meriggi NF, Scacco A, McMurry N, Voors M, et al. COVID-19 vaccine acceptance and hesitancy in low- and middle-income countries. Nat Med. 2021;27(8):1385–94. doi: 10.1038/s41591-021-01454-y 34272499 PMC8363502

[28] Lee H, Noh EB, Park SJ, Nam HK, Lee TH, Lee GR, et al. COVID-19 Vaccine Perception in South Korea: Web Crawling Approach. JMIR Public Health Surveill. 2021;7(9):e31409. doi: 10.2196/31409 34348890 PMC8428376

[29] Jiang LC, Chu TH, Sun M. Characterization of Vaccine Tweets During the Early Stage of the COVID-19 Outbreak in the United States: Topic Modeling Analysis. JMIR Infodemiology. 2021;1(1):e25636. doi: 10.2196/25636 34604707 PMC8448459

[30] Gligorić K, Anderson A, West R. How constraints affect content: The case of Twitter’s switch from 140 to 280 characters. In: Proceedings of the International AAAI Conference on Web and Social Media, 2018.

[31] Charles-Smith LE, Reynolds TL, Cameron MA, Conway M, Lau EHY, Olsen JM, et al. Using Social Media for Actionable Disease Surveillance and Outbreak Management: A Systematic Literature Review. PLoS One. 2015;10(10):e0139701. doi: 10.1371/journal.pone.0139701 26437454 PMC4593536

[32] Yanow D. Seeing Organizational Learning: A `Cultural’ View. Organization. 2000;7(2):247–68. doi: 10.1177/135050840072003

[33] Yanow D. Conducting Interpretive Policy Analysis. Thousand Oaks, California: SAGE Publications. 2000.

[34] Babones S. Interpretive Quantitative Methods for the Social Sciences. Sociology. 2015;50(3):453–69. doi: 10.1177/0038038515583637

[35] Woolf NH, Silver C. Qualitative analysis using NVivo: The five-level QDA method. Routledge. 2017.

[36] Fiesler C, Proferes N. “Participant” Perceptions of Twitter Research Ethics. Social Media + Society. 2018;4(1). doi: 10.1177/2056305118763366

[37] Chen E, Lerman K, Ferrara E. Tracking Social Media Discourse About the COVID-19 Pandemic: Development of a Public Coronavirus Twitter Data Set. JMIR Public Health Surveill. 2020;6(2):e19273. doi: 10.2196/19273 32427106 PMC7265654

[38] Sousa‐Pinto B, Jankin S, Vieira RJ, Marques‐Cruz M, Fonseca JA, Bousquet J. English tweets on allergy: content analysis and association with surveillance data. Clinical & Experimental Allergy. 2024. 10.1111/cea.14479 38567657

[39] Saleh S, McDonald S, Basit M, Kumar S, Arasaratnam R, Perl T. Public perception of COVID-19 vaccines through analysis of twitter content and users. medRxiv. 2021;10. doi: 10.2196/2125570 PMC10288320 37385887

[40] Fiesler C, Proferes N. Participant perceptions of Twitter research ethics. Social Media Society. 2018;4(1):2056305118763366.

[41] Larson HJ, Cooper LZ, Eskola J, Katz SL, Ratzan S. Addressing the vaccine confidence gap. Lancet. 2011;378(9790):526–35. doi: 10.1016/S0140-6736(11)60678-8 21664679

[42] Latkin CA, Dayton L, Yi G, Konstantopoulos A, Boodram B. Trust in a COVID-19 vaccine in the U.S.: A social-ecological perspective. Soc Sci Med. 2021;270:113684. doi: 10.1016/j.socscimed.2021.113684 33485008 PMC7834519

[43] CDC. Building confidence in COVID-19 vaccines. Centers for Disease Control and Prevention. https://www.cdc.gov/vaccines/covid-19/vaccinate-with-confidence.html. 2021. 2021 October 5 34009769

[44] CDC. COVID Data Tracker 2021: Centers for Disease Control and Prevention. 2021. 2021 October 07 https://covid.cdc.gov/covid-data-tracker/#vaccine-confidence.

[45] Daly M, Jones A, Robinson E. Public trust and willingness to vaccinate against COVID-19 in the US from October 14, 2020, to March 29, 2021. JAMA. 2021;325(23):2397–9. 34028495 10.1001/jama.2021.8246 PMC8145162

[46] Pelčić G, Karačić S, Mikirtichan GL, Kubar OI, Leavitt FJ, Tai MC. Religious exception for vaccination or religious excuses for avoiding vaccination. Croatian medical journal. 2016;57(5):516. 27815943 10.3325/cmj.2016.57.516 PMC5141457

[47] Philadelphia TCoPo. Cultural perspectives on vaccination. The College of Physicians of Philadelphia. 2018. https://historyofvaccines.org/vaccines-101/ethical-issues-and-vaccines/cultural-perspectives-vaccination

[48] Ruijs WLM, Hautvast JLA, Kerrar S, van der Velden K, Hulscher MEJL. The role of religious leaders in promoting acceptance of vaccination within a minority group: a qualitative study. BMC Public Health. 2013;13:511. doi: 10.1186/1471-2458-13-511 23711160 PMC3668146

[49] Ruijs WLM, Hautvast JLA, van Ijzendoorn G, van Ansem WJC, van der Velden K, Hulscher MEJL. How orthodox protestant parents decide on the vaccination of their children: a qualitative study. BMC Public Health. 2012;12:408. doi: 10.1186/1471-2458-12-408 22672710 PMC3434025

[50] Shelton RC, Snavely AC, De Jesus M, Othus MD, Allen JD. HPV vaccine decision-making and acceptance: does religion play a role?. J Relig Health. 2013;52(4):1120–30. doi: 10.1007/s10943-011-9553-x 22076049 PMC4616263

[51] UNICEF. Zimbabwe’s religious leaders increase efforts to tackle COVID-19 and support vaccines. https://www.unicef.org/zimbabwe/press-releases/zimbabwes-religious-leaders-increase-efforts-tackle-covid-19-and-support-vaccines. 2021. 2021 September 15

[52] Oyo-Ita A, Bosch-Capblanch X, Ross A, Oku A, Esu E, Ameh S, et al. Effects of engaging communities in decision-making and action through traditional and religious leaders on vaccination coverage in Cross River State, Nigeria: A cluster-randomised control trial. PLoS One. 2021;16(4):e0248236. doi: 10.1371/journal.pone.0248236 33861742 PMC8051768

[53] Forster T, Heinzel M. Reacting, fast and slow: how world leaders shaped government responses to the COVID-19 pandemic. Journal of European Public Policy. 2021;28(8):1299–320. doi: 10.1080/13501763.2021.1942157

[54] Holmberg C, Blume S, Greenough P. The politics of vaccination: a global history. Manchester University Press. 2017.

[55] Sharfstein JM, Callaghan T, Carpiano RM, Sgaier SK, Brewer NT, Galvani AP, et al. Uncoupling vaccination from politics: a call to action. Lancet. 2021;398(10307):1211–2. doi: 10.1016/S0140-6736(21)02099-7 34537104 PMC8445735

[56] Fonseca EMD, Shadlen KC, Bastos FI. The politics of COVID-19 vaccination in middle-income countries: Lessons from Brazil. Soc Sci Med. 2021;281:114093. doi: 10.1016/j.socscimed.2021.114093 34144480 PMC9188662

[57] Kumar VM, Pandi-Perumal SR, Trakht I, Thyagarajan SP. Strategy for COVID-19 vaccination in India: the country with the second highest population and number of cases. NPJ Vaccines. 2021;6(1):60. doi: 10.1038/s41541-021-00327-2 33883557 PMC8169891

[58] Wood S, Schulman K. Beyond Politics - Promoting Covid-19 Vaccination in the United States. N Engl J Med. 2021;384(7):e23. doi: 10.1056/NEJMms2033790 33406324

[59] Alemi F, Lee KH. Impact of Political Leaning on COVID-19 Vaccine Hesitancy: A Network-Based Multiple Mediation Analysis. Cureus. 2023;15(8):e43232. doi: 10.7759/cureus.43232 37692573 PMC10491458

[60] Bokemper SE, Huber GA, Gerber AS, James EK, Omer SB. Timing of COVID-19 vaccine approval and endorsement by public figures. Vaccine. 2021;39(5):825–9. doi: 10.1016/j.vaccine.2020.12.048 33390295 PMC7744009

[61] Bolsen T, Palm R. Politicization and COVID-19 vaccine resistance in the U.S. Prog Mol Biol Transl Sci. 2022;188(1):81–100. doi: 10.1016/bs.pmbts.2021.10.002 35168748 PMC8577882

[62] Kreps SE, Kriner DL. Model uncertainty, political contestation, and public trust in science: Evidence from the COVID-19 pandemic. Sci Adv. 2020;6(43):eabd4563. doi: 10.1126/sciadv.abd4563 32978142 PMC7577608

[63] Dal Poz MR, Levcovitz E, Bahia L. Brazil’s Fight Against COVID-19. Am J Public Health. 2021;111(3):390–1. doi: 10.2105/AJPH.2020.306122 33566654 PMC7893345

[64] Bernardeau-Serra L, Nguyen-Huynh A, Sponagel L, Sernizon Guimarães N, Teixeira de Aguiar RA, Soriano Marcolino M. The COVID-19 Vaccination Strategy in Brazil-A Case Study. Epidemiologia (Basel). 2021;2(3):338–59. doi: 10.3390/epidemiologia2030026 36417230 PMC9620893

[65] Gramacho WG, Turgeon M. When politics collides with public health: COVID-19 vaccine country of origin and vaccination acceptance in Brazil. Vaccine. 2021;39(19):2608–12. doi: 10.1016/j.vaccine.2021.03.080 33846045 PMC8023202

[66] Neiva MB, Carvalho I, Costa Filho EDS, Barbosa-Junior F, Bernardi FA, Sanches TLM, et al. Brazil: the emerging epicenter of COVID-19 pandemic. Rev Soc Bras Med Trop. 2020;53:e20200550. doi: 10.1590/0037-8682-0550-2020 33111917 PMC7580283

[67] Chatterjee N, Mahmood Z, Marcussen E. Politics of Vaccine Nationalism in India: Global and Domestic Implications. Forum for Development Studies. 2021;48(2):357–69. doi: 10.1080/08039410.2021.1918238

[68] Koller CN, Schwerzmann CJ, Lang ASA, Alexiou E, Krishnakumar J. Addressing Different Needs: The Challenges Faced by India as the Largest Vaccine Manufacturer While Conducting the World’s Biggest COVID-19 Vaccination Campaign. Epidemiologia (Basel). 2021;2(3):454–70. doi: 10.3390/epidemiologia2030032 36417236 PMC9620944

[69] Chavda VP, Vihol DR, Solanki HK, Apostolopoulos V. The vaccine world of COVID-19: India’s Contribution. Vaccines. 2022;10(11):1943. 36423038 10.3390/vaccines10111943 PMC9695423

