# Supplementary material for: Preparing for future pandemics: A qualitative exploration of social media in light of the COVID-19 pandemic and vaccine hesitancy
Source: PLOS Glob Public Health. 2025 Jul 7;5(7):e0004317. doi: 10.1371/journal.pgph.0004317 (PMC12233262; doi:10.1371/journal.pgph.0004317)
Supplement: S1 Table — (DOCX) [file pgph.0004317.s001.docx]

**S1 Table**: Codebook for Preparing for Future Pandemics: A qualitative exploration of social media in light of the COVID-19 pandemic and vaccine hesitancy

| **Code** | **Definitions** | **Example Codes** |
| --- | --- | --- |
| Trust | Public perception of the belief that COVID vaccines will work | “…Hiding the real data of COVID vaccine” |
| Duration of vaccine development (time) | Time-span involved in vaccine development |  |
| Side effects | worry about the present and future side effects of the COVID-19 vaccine | “...see we have to understand this COVID is virus and BCG has the power to fight all viruses if made more powerful without hurting the RNA which this Pfizer vaccine will do.” |
| Transparency in vaccine development |  |  |
| Miscommunication | Misleading information on the implication of COVID-19 vaccination | “Actually, a double dose of [Bacillus Calmette-Guerin] BCG vaccine clubbed in one is the best medicine to fight COVID- 19...see we have to understand this COVID is virus and BCG has the power to fight all viruses if made more powerful without hurting the RNA which this Pfizer vaccine will do.” |
| Fear | **Fear-based vaccine misinformation or speculative fear-mongering** | “What if this COVID vaccine is what starts off a zombie apocalypse?” |
| Vaccine efficacy | Whether developed vaccines will work and be effective against COVID-19 | “There is not enough data about the effectiveness of the AstraZeneca vaccine for patients over 65 years of age.” |
| Politics | Influence of political leaders to influence the administering of COVID-19 vaccines | “If COVID-19 is a hoax made up by Democrats to make Trump look bad (as if...), why are the republicans getting vaccinated against it?” |
| Religious beliefs | Influence of religious groups on taking up COVID-19 vaccination | “The coronavirus is widespread in the world, even with the best vaccines, it will take us years to eradicate the virus if there are no other variants; there are more than seven billion people in the world, and each nation with its methods of fighting the virus; it is better to trust God.” |
