# Supplementary material for: Preparing for future pandemics: A qualitative exploration of social media in light of the COVID-19 pandemic and vaccine hesitancy
Source: PLOS Glob Public Health. 2025 Jul 7;5(7):e0004317. doi: 10.1371/journal.pgph.0004317 (PMC12233262; doi:10.1371/journal.pgph.0004317)
Supplement: S2 Table — (DOCX) [file pgph.0004317.s002.docx]

S2 Table provides the criteria guiding the selection of tweets included and excluded in the analysis.

**S2 Table 2:** Study inclusion and exclusion criteria

| **S/N** | **Inclusion criteria** | **Exclusion criteria** |
| --- | --- | --- |
| 1 | Tweets written within the period December 2020-February 2021 | Exclude tweets not written within the time frame December 2020-February 2021 |
| 2 | Include tweets that discuss COVID-19 vaccination | Exclude tweets not discussing COVID-19 Vaccination |
| 3 | Include tweets only focusing on discussing COVID-19 vaccination in the United States, Brazil, or India | Exclude tweets not focusing on discussing COVID-19 vaccination in the United States, Brazil, or India |
| 4 | Include only English language tweets during the study period | Exclude tweets not written in English language |
